# Supports for Mental Well-Being Valued by Healthcare Workers: Qualitative Analysis of Data From a Canadian Cohort of Healthcare Workers During the COVID-19 Pandemic

**DOI:** 10.1177/10482911251322502

**Published:** 2025-03-04

**Authors:** Shannon Marie Ruzycki, Anil Adisesh, Quentin Durand-Moreau, France Labreche, Tanis Zadunayski, Erica Stroud, Nicola Cherry

**Affiliations:** 1 Department of Medicine, 2129 University of Calgary, Calgary, AB, Canada; 2 Department of Occupational Medicine, 7938 University of Toronto, Toronto, ON, Canada; 3 Department of Occupational Medicine, 3158 University of Alberta, Edmonton, AB, Canada; 4 97890 Robert-Sauve Research Institute for Occupational Health and Safety, Montreal, QC, Canada

**Keywords:** healthcare worker well-being, workplace mental health supports, employee assistance programs

## Abstract

A prospective cohort of 4964 HCWs from four Canadian provinces was established early in the COVID-19 pandemic. Participants were invited to comment about workplace mental health supports at three time points. We performed a thematic content analysis of responses from 1738 participants using the Social Support Behaviour Code framework to categorize barriers to support as informational, tangible, emotional, social, or expressing esteem. Themes were synthesized into suggestions for healthcare organizations to prepare for future crises. Formal and informal peer support, workplace mental health supports, and one-on-one counseling were most often mentioned as valued. Analysis suggested that workplace social networks as a source of support and mental health supports would have been appreciated. HCWs perceived that a lack of tangible workplace supports, such as staffing, compensation, and time off, were barriers to well-being. Medical workplaces could consider the availability of tangible supports in addition to developing formal mental health supports for healthcare workers.

## Introduction

Many studies showed psychological and mental health-related repercussions of the COVID-19 pandemic on healthcare workers (HCWs), ranging from increased anxiety scores, depression, and psychological distress compared to the general population or to prepandemic levels.^1–3^ Medical institutions attempted to provide support, without much guidance or evidence of effectiveness.^4,5^ Most studies of HCWs’ stress or mental health coping strategies during the pandemic focused on personal rather than systems-level interventions,^6–9^ were opinion or narrative guides,^10^ or were cross-sectional rather than interventional or prospective studies.^8,9,11^

In the early months of the pandemic, we established a cohort of Canadian HCWs to assess the impact of working through the COVID-19 pandemic on their health.^12,13^ Analysis of this cohort found that 10% had no access to workplace mental health support and about one-third had used workplace mental health support during the pandemic.^13^ Cohort participants were invited to share open text feedback about the additional supports for mental well-being they would have valued. We aimed to analyze the perceptions of HCWs about workplace mental health supports, as these may guide healthcare organizations to develop more effective supports or adapt current supports to be more acceptable to HCWs.

## Methods

### Overview

Data on mental health supports were collected during three follow-up contacts with members of the cohort of Canadian HCWs.^12,13^ Ethical review was provided by the University of Alberta's research ethics board (Pro00009970). Seed funding was provided by the College of Physicians and Surgeons of Alberta. Grant funding was obtained from the Canadian Institutes of Health Research (FRN 173209) and extended by a grant from the Canadian Immunology Task Force.

### Participants and Procedures

A detailed description of the recruitment and demographics of this cohort is available elsewhere.^12^ In brief, HCWs in four Canadian provinces (Alberta, British Columbia, Ontario, and Québec) were invited to participate in a prospective cohort study in spring 2020 through an e-mail invitation from their professional regulatory associations or federations or, in Quebec, through the College of Physicians website. Recruitment included nurses (registered and psychiatric; RNs), licensed practical nurses (LPNs), physicians (MDs), healthcare aides (HCAs), and personal support workers (PSWs). Physicians were recruited in all four provinces, PSWs were recruited only in Ontario, and RNs, LPNs, and HCAs were recruited only from Alberta.

Consenting participants were asked to complete an online questionnaire (Qualtrics, Provo, UT) at four time points during the COVID-19 pandemic: Phase 1 (enrollment and collection of demographic information, from April 2020), Phase 2 (October 2020), Phase 3 (May 2021), and Phase 4 (May 2022). These questionnaires collected information on workplace and work characteristics (such as hospital unit or availability of personal protective equipment), infection with SARS-CoV-2, and vaccination for COVID-19. Questionnaires were available in English or French. Cohort participants were also invited to participate in two substudies as the pandemic progressed: one to evaluate response to vaccination through donation of successive blood samples to determine antibodies to SARS-CoV-2^14^ and the other a case-referent study to identify workplace factors associated with COVID-19 infection.^15^

### Measures

Participants were asked, in Phase 2, Phase 3, and Phase 4 questionnaires, about workplace mental health supports that were available and whether they had used each support. They were asked to indicate availability and use for each of (1) one-on-one support from a specialist counselor, psychologist, or similar; (2) one-on-one support from a colleague or a peer nominated to do this; (3) any online support group where you could discuss and ask questions; (4) an online self-learning tool with advice about how to manage stress; (5) a helpline with a number you could call if you were distressed; (6) an Employee Assistance Program (EAP); (7) or any other mental health support (with an open text option).^13^ Participants were then asked, “Are there other mental health supports you would have valued during this time?,” as an open text response.

### Analysis

Open-text responses to the question about other mental health support that the participant would have valued were coded by two study team members (S.M.R. and E.S.) using thematic content analysis.^16^ S.M.R. is a general internist who worked clinically with patients with COVID-19 infection in Alberta and held leadership positions related to physician wellness during the pandemic. E.S. is a research assistant with experience in qualitative methods. Responses in French were translated into English by a native French speaker before analysis.

Initial codes were developed using the list of possible supports in the original survey questions and through a close reading of all responses by both readers. Codes were refined through discussion between readers and a final codebook was created (eTable 1). Coding was then carried out independently by each reader and all responses were compared for agreement. Discrepancies were resolved by a third member of the research team. Each open-text response was coded to reflect the source of support (related to the workplace, government, or personal life) and the type of support (derived from the questionnaire options and through inductive analysis). Each response was also coded according to its reported effect on well-being, hereafter called “valence of support”; whether the support was anticipated to act as a barrier to well-being, as a facilitator of well-being, or was a suggested support that would have been valued. A single response could have multiple codes on each aspect (e.g., be related to both work and government). Responses that described participant requests for workplace mental health supports were synthesized as suggestions to healthcare organizations looking to improve workplace mental health supports.

Codes that described barriers to mental well-being or engagement with mental health supports were grouped using the constructs of the Social Support Behaviour Code (SSBC). The SSBC was developed in 1990 by Cutrona and Suhr to explore the types of support offered during a discussion of a problem between spouses.^17^ The five categories of support in the SSBC are: informational (information about the stressful event and/or coping with the event), tangible (also called “instrumental”: providing goods or services to assist with the event), emotional (expressing caring), social network (facilitating belonging or community with others experiencing stress), and esteem support (expressing respect and confidence).^17^ Quotations within each SSBC category were then compared for similarities, differences, and patterns to generate theme statements to describe workplace supports for HCWs. These themes tell a core, interpretative story about the support categories from the perspective of HCWs during the COVID-19 pandemic.^18^ Quotations were corrected for grammar and spelling only and were de-identified before presentation in the results.

## Results

The demographics of the 4964 HCWs enrolled in the full cohort are described elsewhere.^12^ This analysis focuses on the 2714 responses from 1738 HCWs to the open text question about which additional supports would have been valued (*n* = 948, *n* = 887, and *n* = 879 responses at Phases 2, 3, and 4, respectively). Most comments were contributed by female participants (*n* = 2437, 89.8%) and RNs (*n* = 1873, 69.0%; Table 1), reflecting the composition of the whole cohort. Of all comments, 89 (3.3%) were noncodable (for example “unsure” or “anything”). Overall, most responses related to the participant's workplace (*n* = 1716, 63.2%; eTable 1). Most facilitators were at the personal level (*n* = 207, 74.5%; e.g., friends, family, exercise) in contrast to only 65 comments mentioning work-related facilitators.

**Table 1.** Demographics of Participants Who Provided a Response to the Open Text Question “Are There Other Mental Health Supports You Would Have Valued During This Time?.”

Demographics	Participants *n* (%)	Comments *n* (%)
Total	1738	2714
Gender		
Female	1549 (89.1)	2437 (89.8)
Male	185 (10.6)	270 (9.9)
X	4 (0.2)	7 (0.3)
Race and/or ethnicity		
Asian	156 (9.0)	230 (8.5)
Black	17 (1.0)	25 (0.9)
Indigenous	44 (2.5)	75 (2.8)
Latin American	13 (0.7)	16 (0.6)
White	1449 (83.4)	2294 (84.5)
Multiracial	16 (0.9)	23 (0.8)
Unknown	43 (2.5)	51 (1.9)
Occupation		
RN	1190 (68.5)	1873 (69.0)
MD	430 (24.7)	663 (24.4)
PSW	55 (3.2)	80 (2.9)
LPN	34 (2.0)	55 (2.0)
HCA	29 (1.7)	43 (1.6)
Age		
<40 years	732 (42.1)	1127 (41.5)
40 to <55 years	632 (36.4)	1010 (37.2)
55 years or older	374 (21.5)	577 (21.3)
Place of work		
Community	687 (39.5)	1134 (41.8)
Hospital	1049 (60.4)	1577 (58.1)
Unknown	2 (0.1)	3 (0.1)

HCA = healthcare aides; LPN = licensed practical nurses; MD = physicians; PSW = personal support workers; RN = registered and psychiatric nurses.

### Requests for Support

We identified six codes to categorize the additional workplace supports that would have been valued by participants from the open-text questions (Table 2). Formal and informal peer support (*n* = 309), characteristics of mental health supports (*n* = 308), and one-on-one counseling (*n* = 240) were most often mentioned by participants (Table 2). Within each category, we created subthemes, phrased as suggestions to healthcare organizations, to describe the facilitators of well-being and suggested improvements to each workplace mental health support as reported by participants (Table 2). The number of comments that mentioned each workplace support generally did not differ by study phase (eTable 2), participant gender (eTable 3), or race and/or ethnicity (eTable 4), with some exceptions. Mental health supports were requested more among female HCWs than non-females (18.6% vs 11.5%; *p* = .03), whereas comments related to HCW skills or knowledge were mentioned by a higher proportion of non-female HCWs than females and HCWs overall (6.4% vs 2.5%; *p* = .01). Requests for hazard or overtime pay differed by participant race and/or ethnicity (*p* = .07) and were mentioned more often by Indigenous (7.9%), Black (8.3%), and multiracial (7.1%) HCWs. Similarly, paid sick or vacation days differed among racial and/or ethnic groups (*p* = .04), with Asian (18.1%) HCWs mentioning this support more often than other HCWs.

**Table 2.** Valued Workplace Supports From Canadian Healthcare Workers During the COVID-19 Pandemic, Paired With Summary Suggestions for Healthcare Organizations.

Supports	Suggestions to Healthcare Organizations	Examples of Participant Quotations
Formal and informal peer support *n* = 309	Facilitate workplace social networks as a source of support.	“I think it would have been beneficial to have some sort of online multidisciplinary support network. To encourage positive coping, hav[ing] people to talked to [who are not on] your unit, providing [information] about coping,” *RN-0173, female, Phase 2* “Drop-in peer support groups to discuss specific challenges pertaining to [my clinical area],” *MD-0040, female, Phase 4* “More freedom at work to socialize with coworkers during work like we used to before pandemic,” *MD-0241, female, Phase 4* “Physician lounge to share experiences with other doc[tor]s,” *MD-0284, male, Phase 2* “Working out in the community is lonely. I'm lucky to see a co-worker in passing about once a week and only go into the office for supplies every 3 or 4 weeks,” *PSW-0435, female, Phase 2* “My workplace has a hiking group … It is a great mental health support, although informal…This is a time [when] we can discuss shared difficulties and little wins experienced at work,” *RN-0633, female, Phase 4*
Workplace-specific? mental health supports *n* = 308	Target or adapt resources for healthcare worker roles or settings.	“Mental health support for male family docs is close to zero,” *MD-0014, female, Phase 4* “I work on a mental health/crisis team—EFAP is inadequate,” *RN-1295, female, Phase 4* “I have used some of the apps available for general stress relief, and would have liked some to be more health worker focused through employer/union,” *RN-5551, female, Phase 2* “I think all health care workers who are also parenting would have benefitted from coordinated support,” *MD-0106, female, Phase 4*
	Ensure supports are available at a range of settings and times.	“Zoom meetings for mental health support that were at more convenient hours,” *MD-0147, female, Phase 3* “Maybe scheduled mental health supports that can be accommodated during work without having to get approval to attend,” *RN-0857, female, Phase 4* “Someone to support staff in-person, in the building,” *RN-5965, female, Phase 4*
	Improve access to existing supports.	“I think a dedicated mental health support line for health care workers would have been ideal, but if this was available in [my province], I never heard about it,” *MD-0553, female, Phase 2* “It would be nice if EFAP returned phone calls…. I called 3 times to try and seek resources for different things and left a message with the triage line and never heard back,” *RN-0233, female, Phase 4* “I feel as though [our provincial health service] may have provided some supports, but they are not easy to find or access. I believe my management could have done a better job at identifying needs on the unit and providing some sort of support,” *RN-1241, female, Phase 3*
One-on-one counseling *n* = 240	Address perceived lack of confidentiality and/or stigma (a barrier to accessing workplace therapists).	“I would have valued the option to actually seek my own counselling services, independent of my organization - rather than through my organization - stigma limits us when they are through work,” *MD-0339, female, Phase 4* “Individual support that is confidential; worried that none of the supports are confidential,” *MD-0426, female, Phase 2* “I pay for my own psychologist… EFAP records and gives information to [our]employer. Myself and my colleagues cannot risk this,” *RN-1077, female, Phase 3*
	Increase the number of sessions offered (often inadequate).	“Easier access to psychologists that we can actually build a rapport with rather than the 3 sessions that the EAP offers,” *RN-0037, female, Phase 2* “Covered, ongoing counselling would be helpful to deal with the anxiety. I have been unable to find counselling for free after EFAP sessions were used,” *RN-5639, female, Phase 3*
	Ensure that therapists are qualified for the needs of HCWs.	“Therapists who actually understood nursing or had worked as a nurse in the past,” *RN-1002, female, Phase 4* “Therapy for therapists (specialized support for those of us working as nurses in mental health, specifically those of us trained as therapists - to have therapy available specific to our needs),” *RN-1858, female, Phase 3* “In person support by a counsellor who is paid more than $25/hr. Quality over quantity,” *RN-6277, female, Phase 4*
Skills *n* = 49	Provide HCWs with education on specific skills needed to work during the COVID-19 pandemic.	“Better technical support and instruction to alleviate the stress produced by all the changes involved in adjusting to video interviews, remote interviewing and learning new technology to do work differently,” *RN-1922, female, Phase 3* “People to come check and teach proper donning and doffing for all staff,” *RN-1514, female, Phase 2*
	Provide HCWs with education on specific coping skills to work during the COVID-19 pandemic.	“Maybe unit learning days with discussions and stress management techniques,” *RN-1256, female, Phase 3* “Training for managers and site directors on how to support their employees,” *RN-1233, female, Phase 4* “Lunch time workshops on coping,” *RN-1158, female, Phase 2* “I know what good sleep hygiene looks like, but I don’t know how to go back to sleep after being woken by a night terror related to working during covid. I know how to maintain boundaries, but I don’t know how to say no to something knowing patients will suffer and die. I know how to have a conversation with differing points of view; I don’t know how to talk to coworkers who chose not to get vaccinated and have no empathy or awareness of the deaths I watched happen with limited ability to help or support, or to ease their suffering as people died without family around because of fear and restrictions. I don’t know how to be neutral or non-confrontational about this topic,” *RN-1292, female, Phase 4*
Online modules *n* = 21	Offer specific online modules (helpful for some healthcare workers).	“I use CALM. Mindfulness, meditation, calming sounds, sleep stories - all helpful,” *RN-1774, female, Phase 4* “The hospital subscribed to Mindwell U last year, an app/program which helped with stress management. It was useful. It would be an asset to have ongoing support from something as practical and time efficient as this,” *MD-0266, female, Phase 2*
Other suggestions *n* = 125	Solicit feedback to understand context-specific supports.	“A place or ability to have uninterrupted quiet time - even a few minutes - to either practice meditation or just be alone,” *RN-0089, female, Phase 4*
		“I would have really appreciated emotional support through a pet therapy program. We had a long-term patient who every day was visited by a family member who had an emotional support dog. I can't tell you how much that helped spending just a brief time with that loving animal!” *RN-1632, female, Phase 4* “Healthier food choices in vending machines when stuck at work unexpectedly,” *RN-4917, female, Phase 2*

EFAP = Employee and Family Assistance Program; HCA = healthcare aide; HCW = healthcare worker; MD = physician; PSW = personal support workers; RN = registered nurse.

Many HCWs reported that they found existing structures to facilitate informal or formal peer support as valuable, including arrangements outside of formal peer support programs such as physical gathering spaces, social activities, or online groups. Many shared that “staying in touch with supportive colleagues” and “just sharing and being able to discuss with colleagues and debriefing at the end of each day” were key supports during the pandemic (*RN-5509, female, Phase 3* and *RN-2053, female, Phase 2*). Conversely, the inability to connect with colleagues was seen by respondents to worsen HCWs’ well-being. This was often mentioned in association with social distancing requirements and other public health measures, and it did not demonstrably change by the study phase.

HCWs also wanted workplace mental health supports that were targeted to their practice setting or role, were available in a range of formats and times, and were more accessible. HCWs reported important barriers to one-on-one counseling programs offered through their workplaces, including a perceived lack of confidentiality, an inadequate number of available sessions, and a sense that available therapists did not have the necessary experience with HCWs. Less commonly, participants requested that their organizations teach pandemic-specific skills such as technical skills and coping strategies.

### Barriers to Mental Well-Being

Participants’ responses that were coded as barriers to using existing mental health supports were grouped into SSBC categories (Table 3). We then wrote theme statements based on quotations in each category (Table 4). Overall, these theme statements suggest that lack of tangible workplace supports were considered an important barrier to well-being for HCWs and that ineffective communication contributed to the perceived lack of informational, emotional, and esteem support (Table 4).

**Table 3.** Demonstration of Categorization of Barriers to Well-Being or Use of Mental Health Support, With Exemplar Quotations From Participants.

Support code (*n*)	Example barrier	SSBC support category
Mental health support (*n* = 308)	“Although supports were offered, they were substandard and hard to access due to a large volume of people, so I gave up trying,” *RN-1865, female, Phase 4*	Tangible
One-on-one counseling (*n* = 240)	“Many of us were/are too exhausted and overwhelmed to initiate finding and making arrangements for counselling appointments on our spare time,” *RN-1376, female, Phase 4*	
Staffing (*n* = 191)	“Lack of resources, lack of funding, [and] lack of adequate staff are the problems we face. Mental health resources for staff through EAP and online stress management will only go so far when it feels like a sinking ship,” *RN-2058, female, Phase 3* “I have a manager who asked me to delay my COVID-19 vaccine booster so that I could work as they would be short staffed that day,” *RN-0177, female, Phase 2*	
Sick days/vacation days (*n* = 184)	“[I would have appreciated the] ability to take some extra time off during lulls in the infection rates in order to get back some balance and perspective/have time to get supports from our families and communities,” *RN-1376, female, Phase 3*	
Time (*n* = 94)	“[I needed] actual time to participate in online support offer(ed) by the union…and not be denied time off to attend [due to] chronic short staffing,” *RN-5461, female, Phase 3*	
Hazard/overtime pay (*n* = 40)	“Better pay for phone calls so I can be safe with patients while not doing free work,” *MD-0133, female, Phase 2* “I work in management normally so while doing contact tracing, other nurses were earning double time while I wasn't even paid straight time for doing the same work. I have never felt more exploited in my career. My mental health was affected but EAP can't fix this,” *RN-0545, female, Phase 2*	
Skills (*n* = 49)	“I am a newly recruited nurse that just started working in an inpatient hospital setting since May 2020. My work orientation was condensed to only 3 days and [I] was placed on a busy unit that also has covid-19 patients. I wish I had a little more training and support from the unit to help integrate as a new nurse,” *RN-0996, female, Phase 2* “It was very stressful not having easy access to the data that explained why client's need not worry about infertility, auto-immune disease, etc, from the COVID vaccines and why they should want to protect themselves. Much of my stress came from…trying to sift through and read the current papers, so I had answers on my next shift,” *RN-5636, female, Phase 4*	Informational
Communication (*n* = 516)	“My immediate leader and my hospital were completely unsupportive and in fact made my job more difficult with vague and contradictory guidance,” *MD-0129, male, Phase 4* “Transparency about where and how COVID infections were transmitted in hospital settings. Only rumors or news outlets gave any information,” *RN-5532, female, Phase 2*	
	“I did not see any unit managers for basically a year. They…never set foot in the clinical area. I wished that a manager could have once a week given us an update in person and gave professional support to the staff,” *RN-0583, female, Phase 3*	Emotional
	“Better day to day recognition from immediate management, to express gratitude more readily and be more transparent to the frontline staff on day-to-day challenges and changes,” *RN-5099, female, Phase 3* “More management involvement and ‘checking in’ to see how we are coping and whether or not we are managing with the new roles and work schedules that we were redeployed to,” *RN-1562, female, Phase 2*	Esteem
Formal and informal peer support (*n* = 309)	“A designated peer support worker… would be helpful. Basically, we all support one another and that's the best we have, but I am concerned as we start to burn out and lack the ability to be a support person for others,” *RN-3506, female, Phase 2*	Social network

EFAP = Employee and Family Assistance Program; HCA = healthcare aide; HCW = healthcare worker; MD = physician; PSW = personal support workers; RN = registered nurse; SSBC = Social Support Behaviour Code.

**Table 4.** Gaps in Workplace Mental Health Support for Canadian Healthcare Workers During the COVID-19 Pandemic.

Support category	Theme(s)	Exemplar quotations
Tangible: goods or services to assist with the event	A lack of time, staffing, sick days, and compensation directly contributed to worse mental health.	“Increase staffing numbers to accommodate the increased time needed to…do appropriate education for my patients. Instead, we encountered staff cuts and a decrease in staff skill sets…it has created a very discontent staff team and very unsatisfying work. Many RNs I know personally are looking for ways to get out of this career,” *RN-5617, female, Phase 3* “[Our provincial health authority] didn’t even bother to give us the $2.00 top up [in salary] as they didn’t think we rated that! How do you think that makes us feel? We work our butts off to make sure our clients [and] co-workers are safe. Last to be provided with masks, no face shields, and now a glove shortage. I love my job, but we deserve more respect,” *HCA-0016, female, Phase 2*
	A lack of time, staffing, sick days, and compensation prevented healthcare workers from using other workplace supports.	“Modules [about] resiliency and stress reduction are not worth much, and are even counterproductive and feel somewhat condescending. What we needed and need is more staff and more space to deal with the deluge of patients…having to do a job when understaffed and under resourced…takes a toll that no web-based seminar squeezed in between the rush of patients is going to accomplish. That management believed such things were adequate feels a bit offensive and makes one wonder about their awareness and grasp of the scope of the problem,” *MD-0185, male, Phase 4* “Online course managing stress might have helped, but…the working conditions have dramatically declined in retirement homes. PSWs are routinely getting 17 or more patients up for breakfast in a two-hour period, then returning in the evening to put patients to bed. Patient care is fast and rough. The idea of patient-centered care is nonexistent,” *PSW-0126, male, Phase 2* “I don’t have time for self-care. The financial impact of virtual care leaves me working longer for less money,” *MD-0550, female, Phase 2*
Informational: information about the event or about coping with the event	Gaps in pandemic-related skill development contributed to stress at work.	“My anxiety comes from seeing others mishandle PPE as in masks and gloves,” *PSW-0282, female, Phase 3* “We were sent to ICU in March, April, & May to be buddied, so we could learn to care for COVID patients if the numbers expanded … I’m 62, & had no intention in my career to work ICU…I don’t know what support they could offer us, but there is unbelievable stress,” *RN-2164, female, Phase 2*
	A lack of clear communication from managers/leaders contributed to stress at work.	“If the pandemic was discussed openly and a plan of action made immediately, it would have helped my mental health - however there seemed to be no cohesive plan, just ‘winging it’,” *RN-0880, female, Phase 2* “When one of our staff contracted COVID, the communication wasn't handled well, and it caused a lot of gossip, anger, frustration, and staff bickering that could've been prevented by better communication and sharing of concerns by frontline workers,” *RN-1589, female, Phase 2*
Emotional: expressing caring	Communications from managers/leaders did not provide adequate emotional support.	“We lost one of our staff and a [family member] of a staff member and not once [did] our manager offer support to our team. It has taken its toll on us, and now we find out that of our team of 6, we are all on antidepressants and are attending counselling separately… our manager should have shown support to us in this time of need,” *RN-0559, female, Phase 4* “It would be nice to have people acknowledge how much extra stress COVID has brought into the workplace, and provide us with support options including group discussions, who we could reach out to, etc,” *RN-6300, female, Phase 2*
Esteem: expressing respect and confidence in abilities to cope	Communications from managers/leaders did not convey respect.	“Basic respect would be valued… the ‘mental health supports’ that [my health organization’ offers are just for show. When it comes down to it, you are just a number,” *RN-0880, female, Phase 3*
Social network: facilitating belonging or community with others who may have similar experiences	Workplace pandemic restrictions were a barrier to social network supports.	“It was very isolating to have to do our jobs and then not have the ‘hallway’ talks we used to. It's strange but these were such a source of support and coping…We [can’t] even sit beside a co-worker without social distancing,” *RN-0061, female, Phase 4*
	Public health pandemic restrictions were a barrier to social network supports.	“[I would have liked] going to meet colleagues for dinner to discuss (but) everything was closed,” *MD-0499, female, Phase 2*

Inadequate staffing, a lack of hazard or overtime pay, and insufficient time off work (either sick days or vacation days) were participant-stated gaps in tangible support for each healthcare occupation in the study (Table 4). RNs, PSWs, and HCAs shared that “staffing ratios are completely unsafe,” (*RN-2336, female, Phase 3*). For physicians, the inability to take time off work due to a lack of staff prevented participants from “(having) time for personal relaxation, exercise, reflection, sleep, time in nature,” (*MD-0292, female, Phase 3*) to address mental health. Many participants reported that they were unable to take their vacation time: “I worked for 15 months straight with only 3 days completely off at Christmas” (*MD-0363, female, Phase 3*).

Lack of informational support due to communication gaps within health organizations was also a central theme, mentioned in 30% of all open text responses by respondents from all healthcare roles. Responses called for “actual experts giving us confidence in what we are doing,” or “clear and concise direction in a timely fashion from a governing body” (*PSW-0116, female, Phase 2* and *MD-0390, female, Phase 2,* respectively). Similarly, participants reported learning more about “outbreaks at (my) place of work, cancellations of surgeries, etc., from the 6 o’clock news” than from “management, (who) kept employees in the dark…(which) increased stress!” (*RN-1740, female, Phase 2*).

In addition, lack of communication within health organizations contributed to gaps in emotional and esteem supports. Calls for “more support from management” were common from RNs, PSWs, LPNs, and HCAs. One RN shared that her “direct supervisor/manager never once asked how I am doing working through the pandemic,” (*RN-3059, female, Phase 2*), and others shared that they “would’ve valued just being heard in some way. Hearing (that) help will come, or this will pass… when you have questions or are frightened… (rather than) getting a response from your employer that this is the job you chose, and you should’ve understood the possible risk” (*PSW*-*0066, male, Phase 4*). In particular, participants perceived a lack of emotional support from managers and leaders who communicated by e-mail rather than in-person, which they felt was “impersonal and heartless” (*RN-1068, female, Phase 2*) and contributed to feeling “terribly alone” (*RN-1476, female, Phase 2*).

## Discussion

This qualitative analysis of survey responses about workplace supports valued during the COVID-19 pandemic among a Canadian cohort of HCWs aims to provide some guidance to healthcare organizations in preparation for future health crises. The results suggest that healthcare organizations could facilitate workplace social networks as a source of support, improve existing mental health supports by increasing accessibility, and invest in therapy programs with counselors who have experience in healthcare. The frequent mention of peer support suggests that healthcare organizations should not overlook methods of facilitating peer support when designing strategies to support HCW well-being. Furthermore, the use of an existing framework of supports in interpersonal relationships^17^ allowed us to examine further barriers to well-being or using support for HCWs.

In Canada, provincial and territorial governments are responsible for regulating occupational hazards, including psychosocial hazards. There are several Canadian^19,20^ guidance documents that can be used to help address psychosocial hazards in workplaces. The Canadian National Standard on Psychological Health and Safety in the Workplace^20^ provides an organizational approach to psychosocial occupational hazards, outlining 13 workplace factors including psychological and social supports (such as the tangible supports we have outlined here) but also psychological demands, recognition, and organizational culture. Recently developed international frameworks^21^ can also provide a structure to address psychosocial occupational hazards. For example, the International Labor Organization Convention 190 on violence and harassment has been ratified by Canada and has been in force since January 2024.^22^

However, even with “hard-law” requirements for occupational health and safety, there are challenges with implementation. The lack of occupational medicine specialists and experts within Canadian healthcare organizations may mean that such resources are not necessarily well-known and used in practice by healthcare organizations. Concerns about the lack of enforcement and the lack of effectiveness of workplace regulation in Canada have been raised elsewhere.^21^ While inspections associated with penalties have been proven very effective in the prevention of occupational injuries,^23^ McLeod et al.^24^ suggest that inspections alone may be insufficient to reduce work injuries. Workers, when asked directly, are unlikely to mention such inspections as resources. In our study, the government was mentioned by less than 5% of our sample as a facilitator or barrier to well-being support, suggesting that many Canadian HCWs have not considered the potential role of regulation in improving workplace conditions. Similarly, our participants named mental health supports as valued support, with few mentioning organizational change. This result may reflect workers’ belief that they are unable to change the way in which work is organized. Their failure to spontaneously report the need for such changes cannot be interpreted as evidence that all is well.

The implementation of specific legislation by provinces may not be sufficient to tackle all aspects of psychosocial workplace hazards. Research conducted in the European Union by Jain et al.^25^ showed that specific legislation requiring workplaces to prevent psychosocial risks led to more companies creating work-stress reduction plans. Unfortunately, this same analysis demonstrated that most companies increased individual-level resources rather than using organizational-level interventions such as restructuring work to reduce job demands.^25^ From our analysis presented here, we report that a lack of tangible support was considered a barrier to engagement with other well-being supports by 18% of open-text respondents. This finding implies that specific programs to improve HCW well-being should be complemented by adequate resources to perform work. We must be clear that the reduction of psychosocial risks in workplaces should not be dealt with only by providing support, but also by modifying the work organization itself and its demands. Altogether, we suggest that effective implementation of legislation to reduce occupational psychosocial hazards for HCWs will require provincial governments to coordinate this legislation with resources to enforce legislation and reduce job demands for HCWs.

Our findings are also consistent with those from a meta-synthesis of qualitative studies examining the experiences of HCWs during the COVID-19 pandemic,^26^ which found that insufficient resources, an unmet need for rest, and poor communication were common across multiple countries and healthcare settings. These results back the conclusions of David et al.^4^ and Labrague et al.^9^ that healthcare organizations must embed their mental health support services within tangible resources to meet the personal and professional needs of HCWs. Despite this, many of the interventions described in the literature that aimed to improve HCW well-being during the pandemic have not included tangible supports.^11^ Tangible supports reported in the literature that may be helpful for HCWs in the COVID-19 pandemic include childcare support, food, lodging when in isolation, spaces for rest at work, and training.^4,5,9^ Our data suggest that HCWs also desire adequate workforce, isolation and quarantine pay, vacation and sick days, and compensation for increased workload. Further, we find that certain tangible supports, such as hazard pay and sick days, may be more important to Asian, Black, and Indigenous HCWs than other groups, though this analysis should be considered exploratory as we did not adjust for work role, gender, and other factors that may confound or modify the association between race and/or ethnicity and requests for supports.

This study has several limitations. First, the transferability of conclusions from this analysis of Canadian HCWs to other settings, including future healthcare crises, is not known. Second, the analysis of open-text responses is limited by a lack of depth and follow-up questions, and it is possible that some participants’ responses may have been misinterpreted. Open-text responses are also limited by the potential for response bias: HCWs who had more positive or more negative experiences with workplace mental health supports may be more likely to submit additional, open-text comments (40% of participants provided open-text responses). Desired mental health supports may not have been possible, given the resource constraints or limitations due to public health measures. Further, the cohort comprised few in healthcare occupations other than nurses and physicians, and the results may not apply to all HCWs.

## Conclusion

In this prospective cohort study of HCWs, we report how common workplace mental health supports may not have been sufficient in the pandemic context. Our analysis of comments on supports and barriers to their use highlights some gaps in healthcare organizations’ response to protecting the mental health of HCWs. These suggestions may guide healthcare organizations to adapt or expand mental health supports during healthcare crises. The importance of tangible supports and effective communication should always be given consideration in crises such as the COVID-19 pandemic.

## Supplemental Material

sj-docx-1-new-10.1177_10482911251322502 - Supplemental material for Supports for Mental Well-Being Valued by Healthcare Workers: Qualitative Analysis of Data From a Canadian Cohort of Healthcare Workers During the COVID-19 Pandemic Supplemental material, sj-docx-1-new-10.1177_10482911251322502 for Supports for Mental Well-Being Valued by Healthcare Workers: Qualitative Analysis of Data From a Canadian Cohort of Healthcare Workers During the COVID-19 Pandemic by Shannon Marie Ruzycki, Anil Adisesh, Quentin Durand-Moreau, France Labreche, Tanis Zadunayski, Erica Stroud and Nicola Cherry in NEW SOLUTIONS: A Journal of Environmental and Occupational Health Policy
