# Supplementary material for: Supports for Mental Well-Being Valued by Healthcare Workers: Qualitative Analysis of Data From a Canadian Cohort of Healthcare Workers During the COVID-19 Pandemic
Source: New Solut. 2025 Mar 4;35(2):162–72. doi: 10.1177/10482911251322502 (PMC12222834; doi:10.1177/10482911251322502)
Supplement: sj-docx-1-new-10.1177_10482911251322502 - Supplemental material for Supports for Mental Well-Being Valued by Healthcare Workers: Qualitative Analysis of Data From a Canadian Cohort of Healthcare Workers During the COVID-19 Pandemic [file sj-docx-1-new-10.1177_10482911251322502.docx]

Requests for support for healthcare workers’ mental well-being:

Qualitative analysis of data from a Canadian cohort of healthcare workers during the COVID-19 pandemic

Appendix

| **eTable 1.** Final codebook used to code participant open text responses. | 2 |
| --- | --- |
| **eTable 2.** Number of comments that mentioned each support for healthcare worker well-being by study phase, stratified by whether the comment mentioned the support as a facilitator, barrier, or suggestion. | 3 |
| **eTable 3.** Number of comments that mentioned each support for healthcare worker well-being by gender. | 4 |
| **eTable 4.** Number of comments that mentioned each support for healthcare worker well-being by ethnicity. | 5 |

**eTable 1.** Final codebook used to code participant open text responses.

| **Code** | **Definition** | **Example** | **Number (%)** |
| --- | --- | --- | --- |
| Level of Support | | | |
| Government | Explicitly referring to the provincial government, health authority, or federal government. | “So-called mental health supports are invalid to me. The only viable and useful supports would be… For the [political party] government to cease their threats and their ongoing war against health care workers,”  *RN-0874, male, Phase 2* | 124 (4.6) |
| Personal | Referring to a support that is available outside of work. | “Self-care, for example, massage and exercise,”  *MD-0367, female, Phase 4* | 520 (19.2) |
| Workplace | Referring to a support that was accessed at work or would be accessed through work as a healthcare provider. | “[I appreciated] having the hospital gym reopen. It has always been a major source of stress reduction,”  *MD-0503, female, Phase 2* | 1,716 (63.2) |
| Not codable | Response is not clearly related to government, personal, or workplace source. | “I wish I would have got counselling sooner,”  *RN-2143, Phase 4* | 407 (15.0) |
| Type of Support | | | |
| Online modules | Referring to an online course, module, or program used as a mental health support. | “I am now doing an online trauma program through Sounds True. It is 15 months in duration and excellent,”  *MD-0324, female, Phase 3* | 53 (2.0) |
| One-on-one counselling | Referring to a therapist, psychologist, psychiatrist, or one-on-one counsellor. | “We have a psychologist on the unit for the staff,”  *RN-1027, female, Phase 4* | 240 (8.8) |
| Peer support | Referring to advice, de-briefing, or discussion with a peer (colleague) with shared lived experience. | “Debriefing after crazy shift,”  *RN-1789, female, Phase 3* | 309 (11.4) |
| Mental health support | Referring to “mental health support” explicitly. | “I made extensive use of mental health resources that I accessed privately. I now have a counsellor, a coach and several support groups which have played a huge part in my healing from burnout that led me to leave my practice,”  *MD-0330, female, Phase 4* | 308 (11.3) |
| Staffing | Referring to healthcare worker colleagues available in their workplace. | “Having more nurses on duty to compensate for the significant extra workload of constant donning and doffing of gowns, with most patients on isolation… there is no recognition that this has caused a major increase in the workload,”  *RN-1254, female, Phase 2* | 191 (7.0) |
| Compensation | Referring to compensation for healthcare work. | “[I would have appreciated] assurance of income if I happened to get covid-19 infection,”  *MD-0290, male, Phase 4* | 40 (1.5) |
| Sick/vacation days | Referring to time away from healthcare work. | “I am fortunate that I had enough sick time to cover pay on my days of isolation. Had it been that I didn't have them, it [would have] definitely lower[ed] my income if I [had to] access short term disability,”  *RN-1189, female, Phase 3* | 184 (6.8) |
| Communication | Referring to the methods, amount, or presence/absence of communication related to healthcare work. | “The [provincial medical association] initially was very helpful with its virtual town halls when the pandemic hit. These were very well organized and an excellent resource that helped to reduce my stress,”  *MD-0232, female, Phase 2* | 516 (19.0) |
| Time | Referring to the time used or needed for mental health supports. | “We were too busy to access anything… we are way too overworked and undervalued,”  *MD-0366, female, Phase 4* | 93 (3.4) |
| Skills | Referring to the knowledge or skills needed or desired to work as a healthcare worker. | “[We needed] more clinical nurse educators available on my unit to support staff and help with teaching for ongoing changes and important practices such as PPE,”  *RN-0721, female, Phase 2* | 49 (1.8) |
| Family | Referring to supports directed at a healthcare workers family members | “Something related to how to cope with our children as healthcare workers - their potential increased risk to infect others at school or day home settings,”  *RN-0138, female, Phase 2* | 43 (1.6) |
| Other | Referring to a support that was not covered by another code | “The warehouse system with stocking failed us and the different hand sanitizers and products were inferior and inappropriate at times,”  *RN-0155, female, Phase 2* | 370 (13.6) |
| Not codable | The subject of the participants comment was not clear or was not related to the workplace. | “A psychologically safe workplace,”  *MD-0053, male, Phase 3* | 231 (8.5) |
| Valence of Support | | | |
| Suggestion | The participant described a change to an existing support OR desire for a support that did not exist. | “A designated peer support worker… would be helpful. Basically, we all support one another and that's the best we have, but I am concerned as we start to burn out and lack the ability to be a support person for others,”  *RN-3506, female, Phase 2* | 1,008 (37.1) |
| Barrier | The participant described a factor that prevented them from accessing an existing mental health support OR a factor that prevented a support from working as intended. | “It was very isolating to have to … not have the ‘hallway’ talks we used to. It’s strange but these were such a source of support and coping. At least in these we could see that we were part of something. We [can’t] even sit beside a co-worker without social distancing,”  *RN-0061, female, Phase 4* | 444 (16.4) |
| Facilitator | The participant described a support that improved their mental well-being OR a factor that allowed a support to work well. | “My co-workers made a chat/coffee group after work as many were going though terrible things. We weren't allowed to gather officially but we would stay after work and share food and drinks to support each other. This has carried on in restaurants now, and has been enormously fun,”  *RN-1427, female, Phase 4* | 289 (10.6) |
| Not codable | It was not clear if the participant was making a suggestion or felt that the subject of their comment was a barrier or facilitator to their well-being. | “Family division,”  *RN-0331, male, Phase 2* | 1,014 (37.4) |

**eTable 2.** Number of comments that mentioned each support for healthcare worker well-being by study phase, stratified by whether the comment mentioned the support as a facilitator, barrier, or suggestion.

|  | **Valence^*^** | **Phase 2**  N (%) | **Phase 3**  N (%) | **Phase 4**  N (%) |
| --- | --- | --- | --- | --- |
| Mental health supports | Facilitator | 2 (0.2) | 4 (0.5) | 4 (0.5) |
|  | Barrier | 21 (2.2) | 35 (3.9) | 45 (5.1) |
|  | Suggestions | 56 (5.9) | 46 (5.2) | 45 (5.1) |
| One-on-one counselling | Facilitator | 2 (0.2) | 4 (0.5) | 11 (1.3) |
|  | Behaviour | 11 (1.2) | 19 (2.1) | 20 (2.3) |
|  | Suggestions | 33 (3.3) | 32 (3.6) | 50 (5.7) |
| Staffing | Facilitator | 1 (0.1) | 1 (0.1) | 1 (0.1) |
|  | Barrier | 15 (1.6) | 19 (2.1) | 14 (1.6) |
|  | Suggestion | 34 (3.6) | 39 (4.4) | 54 (6.1) |
| Sick/vacation days | Facilitator | 0 | 3 (0.3) | 2 (0.2) |
|  | Barrier | 5 (0.5) | 5 (0.6) | 6 (0.7) |
|  | Suggestion | 31 (3.3) | 39 (4.4) | 41 (4.7) |
| Time | Facilitator | 0 | 1 (0.1) | 1 (0.1) |
|  | Barrier | 11 (1.2) | 19 (2.1) | 18 (2.0) |
|  | Suggestion | 12 (1.3) | 14 (1.6) | 10 (1.1) |
| Hazard/overtime pay | Facilitator | 0 | 0 | 0 |
|  | Barrier | 5 (0.5) | 1 (0.1) | 2 (0.2) |
|  | Suggestion | 9 (0.9) | 5 (0.6) | 11 (1.3) |
| Skills | Facilitator | 1 (0.1) | 1 (0.1) | 1 (0.1) |
|  | Barrier | 5 (0.5) | 0 | 3 (0.3) |
|  | Suggestion | 14 (1.5) | 10 (1.1) | 6 (0.7) |
| Communication | Facilitator | 8 (0.8) | 2 (0.2) | 1 (0.1) |
|  | Barrier | 37 (3.9) | 35 (3.9) | 31 (3.5) |
|  | Suggestion | 117 (12.3) | 104 (11.7) | 104 (11.8) |
| Peer support | Facilitator | 8 (0.8) | 10 (1.1) | 7 (0.8) |
|  | Barrier | 8 (0.8) | 7 (0.8) | 8 (0.9) |
|  | Suggestion | 43 (4.5) | 41 (4.6) | 35 (4.0) |
| Online modules | Facilitator | 3 (0.3) | 0 | 3 (0.3) |
|  | Barrier | 2 (0.2) | 0 | 3 (0.3) |
|  | Suggestion | 3 (0.3) | 0 | 2 (0.2) |
| **Total** | | **948** | **887** | **879** |

*Not all comments could be coded with a valence (for example, “mental health supports”), and some comments could be coded with more than one valence (for example, “Peer support was helpful but I wish more times had been available”).

**eTable 3.** Number of comments that mentioned each support for healthcare worker well-being by gender

| Support | **Non-Female** | | **Female** | | **Total** | | **P*** |
| --- | --- | --- | --- | --- | --- | --- | --- |
|  | N | % | N | % | N | % |  |
| Online modules | 2 | 1.3 | 19 | 1.2 | 21 | 1.2 | 1.000^a^ |
| One-on-one counselling | 25 | 15.9 | 215 | 13.8 | 240 | 14.0 | 0.463 |
| Peer support | 29 | 18.5 | 280 | 18.0 | 309 | 18.0 | 0.874 |
| Mental health supports | 18 | 11.5 | 290 | 18.6 | 308 | 17.9 | 0.026 |
| Staffing | 16 | 10.2 | 175 | 11.2 | 191 | 11.1 | 0.695 |
| Hazard/overtime pay | 4 | 2.5 | 36 | 2.3 | 40 | 2.3 | 0.781^a^ |
| Sick/vacation days | 16 | 10.2 | 168 | 10.8 | 184 | 10.7 | 0.821 |
| Communication | 43 | 27.4 | 473 | 30.3 | 516 | 30.1 | 0.442 |
| Time | 4 | 2.5 | 90 | 5.8 | 94 | 5.5 | 0.091 |
| Skills | 10 | 6.4 | 39 | 2.5 | 49 | 2.9 | 0.011^a^ |
| **Total** | **157** | **100.0** | **1559** | **100.0** | **1716** | **100.0** |  |

*Pearson Chi-Square Test

^a^ Fisher’s Exact Test

**eTable 4.** Number of comments that mentioned each support for healthcare worker well-being by ethnicity

| Support | **Indigenous** | | **Asian** | | **Black** | | **White** | | **Latin American** | | **Mixed** | | **Unknown** | | **Total** | | **P*** |
| --- | --- | --- | --- | --- | --- | --- | --- | --- | --- | --- | --- | --- | --- | --- | --- | --- | --- |
|  | N | % | N | % | N | % | N | % | N | % | N | % | N | % | N | % |  |
| Online modules | 0 | 0.0 | 2 | 1.4 | 1 | 8.3 | 17 | 1.2 | 0 | 0.0 | 0 | 0.0 | 1 | 3.3 | 21 | 1.2 | 0.241 |
| One-on-one counselling | 7 | 18.4 | 17 | 12.3 | 3 | 25.0 | 211 | 14.3 | 1 | 12.5 | 0 | 0.0 | 1 | 3.3 | 240 | 14.0 | 0.246 |
| Peer support | 8 | 21.1 | 22 | 15.9 | 5 | 41.7 | 269 | 18.2 | 1 | 12.5 | 2 | 14.3 | 2 | 6.7 | 309 | 18.0 | 0.252 |
| Mental health supports | 7 | 18.4 | 26 | 18.8 | 1 | 8.3 | 265 | 18.0 | 2 | 25.0 | 2 | 14.3 | 5 | 16.7 | 308 | 17.9 | 0.979 |
| Staffing | 5 | 13.2 | 13 | 9.4 | 1 | 8.3 | 161 | 10.9 | 0 | 0.0 | 5 | 35.7 | 6 | 20.0 | 191 | 11.1 | 0.088 |
| Hazard/overtime pay | 3 | 7.9 | 4 | 2.9 | 1 | 8.3 | 30 | 2.0 | 0 | 0.0 | 1 | 7.1 | 1 | 3.3 | 40 | 2.3 | 0.067 |
| Sick/vacation days | 1 | 2.6 | 25 | 18.1 | 1 | 8.3 | 150 | 10.2 | 0 | 0.0 | 2 | 14.3 | 5 | 16.7 | 184 | 10.7 | **0.040** |
| Communication | 11 | 28.9 | 35 | 25.4 | 3 | 25.0 | 450 | 30.5 | 4 | 50.0 | 4 | 28.6 | 9 | 30.0 | 516 | 30.1 | 0.775 |
| Time | 3 | 7.9 | 5 | 3.6 | 0 | 0.0 | 84 | 5.7 | 1 | 12.5 | 0 | 0.0 | 1 | 3.3 | 94 | 5.5 | 0.684 |
| Skills | 0 | 0.0 | 6 | 4.3 | 0 | 0.0 | 40 | 2.7 | 0 | 0.0 | 2 | 14.3 | 1 | 3.3 | 49 | 2.9 | 0.211 |
| **Total** | **38** | **100.0** | **138** | **100.0** | **12** | **100.0** | **1476** | **100.0** | **8** | **100.0** | **14** | **100.0** | **30** | **100.0** | **1716** | **100.0** |  |

*Fisher-Freeman-Halton Exact Test of significance (2-sided Monte Carlo)
